# Trends in cannabis urine drug screen testing in Colorado’s largest health system: a retrospective cohort study

**DOI:** 10.1186/s42238-026-00418-8

**Published:** 2026-03-03

**Authors:** Brock J. Gilsdorf, Laura J. Helmkamp, Robert L. Page, Larry A. Allen, Colleen K. McIlvennan

**Affiliations:** 1 Department of Medicine, Internal Medicine Residency Program, University of Colorado School of Medicine, 12631 E 17th Ave, Mailstop B177, CO Aurora, 80045 USA; 2 Adult and Child Center for Health Outcomes Research and Delivery Science (ACCORDS), University of Colorado, Aurora, CO USA; 3 Department of Clinical Pharmacy, University of Colorado Skaggs School of Pharmacy and Pharmaceutical Sciences, Aurora, USA; 4 Division of Cardiology, Department of Medicine, University of Colorado School of Medicine, Aurora, CO USA

**Keywords:** Cannabis, Marijuana, Urine drug screen, Disparities, Colorado, Retrospective, Cohort

## Abstract

**Background:**

Colorado was the first state in the United States to legalize recreational cannabis, which provides a unique opportunity to evaluate cannabis urine drug screen (UDS) testing trends in a large healthcare system in response to that policy. This study examined how testing rates and positivity evolved over time and whether testing patterns varied by demographic factors.

**Methods:**

In this retrospective cohort study, we analyzed all University of Colorado Health visits from 2008 to 2022 using electronic medical record data. We assessed group comparisons with Chi-squared tests and t-tests and demographic predictors of UDS testing using multivariable log binomial models.

**Results:**

From 2008 to 2022, the positivity rate for cannabis among UDSs rose from 14% in 2008 to 30% in 2015, then plateaued. The proportion of visits with cannabis testing declined from 0.25% to 0.04%. Younger patients, males, and individuals identifying as Black, Hispanic, or American Indian were more likely to be tested than their counterparts, with notable racial and ethnic disparities.

**Conclusions:**

Despite rising cannabis positivity rates, testing frequency declined. Persistent demographic disparities in testing raise concerns about potential bias, especially in the absence of documented differences in use across groups. Future work should examine indications for cannabis urine drug screen testing and how results influence clinical care.

**Supplementary Information:**

The online version contains supplementary material available at 10.1186/s42238-026-00418-8.

## Background

Cannabis legalization in the United States has expanded rapidly over the past two decades. Colorado legalized medical cannabis in 2000 and, in 2012, became the first state to legalize recreational cannabis for adult use. Despite these policy changes, there have been limited reports describing cannabis urine drug screen testing practices and test positivity rates across large patient populations, particularly at the level of an entire healthcare system.

Urine drug screen (UDS) testing for cannabis is commonly used in clinical care for a variety of indications and a variety of patient care settings. However, UDS testing does not measure cannabis use prevalence directly; rather, it reflects clinician testing behavior and perceived clinical utility, along with institutional practices. Understanding how cannabis testing practices evolve over time is therefore critical to interpreting UDS results in the context of changing legal and social norms.

Prior studies examining the impact of cannabis legalization in Colorado have largely focused on healthcare utilization and acute clinical presentations. Early reports described increases in emergency department visits related to cannabis-associated conditions such as cyclic vomiting syndrome, pediatric exposures, and burn injuries (Monte et al. 2015; Wang et al. 2013). While these studies provided important insights into the clinical consequences of legalization, they were limited to specific patient populations or clinical syndromes and did not evaluate system-wide cannabis urine drug screen testing practices, test positivity rates, or demographic patterns in testing.

Examination of patient demographics is particularly important given longstanding concerns about inequities in substance-related screening. Previous studies have demonstrated that urine drug testing is not applied uniformly across patient populations in various clinical settings, with documented differences by race or ethnicity (Peterson et al. 2023; Olaniyan et al. 2023; Ortiz et al. 2021). Importantly, population-based studies suggest that differences in cannabis use prevalence do not fully explain these testing disparities (Miech et al. 1975).

Using data from Colorado’s largest integrated healthcare system over a 15-year period spanning pre- and post-recreational cannabis legalization, we sought to characterize longitudinal trends in cannabis urine drug screen testing and test positivity. We additionally examined whether testing rates differed by age, sex, and race/ethnicity. We hypothesized that cannabis UDS positivity would increase over time following legalization, that the frequency of cannabis testing would decline as cannabis use became more normalized, and that demographic disparities in testing would persist despite legalization.

## Methods

The aim of this study was to characterize longitudinal trends in cannabis urine drug screen testing within a large integrated healthcare system. We conducted a retrospective cohort study including all patients within University of Colorado Health (UCHealth), a large non-profit, integrated healthcare system serving urban and rural populations across Colorado, Wyoming, and Nebraska. The study period spanned January 1, 2008, through December 31, 2022, encompassing years before and after legalization of recreational cannabis in Colorado. Electronic medical record (EMR) data were obtained through Health Data Compass, the comprehensive health data hub at UCHealth. Data obtained includes patient demographic data and number of encounters per year for all patients in the UCHealth system over this time period. For patients with UDS testing, laboratory results were also obtained which detects cannabis metabolites of delta-9-tetrahydrocannabinol, most commonly the inactive primary metabolite 11-nor-9-carboxy-delta-9-tetrahydrocannabinol (THC-COOH).

Demographic variables were described using percentages and counts among our patient sample. The percentage of patients in a given demographic category who received a cannabis test during the study period was described using counts and percentages. Multivariable log binomial models at the encounter level were used to obtain adjusted risk ratios (RRs) for cannabis testing at a given encounter, with age, sex, and race/ethnicity included simultaneously as covariates. Subjects missing data for a demographic variable were included in descriptive tables and adjusted models with an ‘Unknown’ category.

In additional models, encounters were divided into three temporal blocks, 2008–2012, 2013–2017, and 2018–2022, and interaction terms between temporal block and age, race/ethnicity, and gender were added to the models. Risk ratios for probability of UDS testing at a given visit by demographic category were compared over time to examine any changes in disparities over time.

All analyses were conducted in SAS version 9.4. Adjusted risk ratios with a 95% confidence interval not containing 1 were considered statistically significant.

This study was reviewed by Colorado Multiple Institutional Review Board and deemed exempt from IRB review.

## Results

A total of 4,205,734 patients visited the UCHealth system between 2008 and 2022 and were included in the analysis. Patients spanned all age groups, with over a third of patients in the 18–39-year age range (33.7%). The majority of patients were female (53.4%), and most patients identified as non-Hispanic white (62.9%) (Table 1). UCHealth primarily serves patients in Colorado, and expanded facilities into Wyoming and Nebraska during the study period; system-level data indicates that approximately 5% of patients served during the study period resided outside of Colorado.

**Table 1:** Association between patient demographics and cannabis urine drug screen testing among UCHealth patients, 2008–2022, from multivariable models

	Patient demographics % (*n*) *n* = 4,205,734	Patients with cannabis UDS, % (*n*)	Adjusted RR (95% CI)
Age			
Under 18	14.1% (592,230)	0.8% (4,834)	5.89 (5.59, 6.20)
18–39	33.7% (1,418,116)	2.1% (30,208)	13.70 (13.11, 14.32)
40–59	24.9% (1,045,271)	1.8% (19,311)	8.63 (8.25, 9.02)
60–79	20.8% (873,743)	1.2% (10,852)	3.78 (3.62, 3.96)
80+	5.0% (211,756)	0.7% (1,422)	- reference -
Unknown	1.5% (64,618)	0.0% (30)	2.42 (1.69, 3.48)
Sex			
Female	53.4% (2,242,139)	1.6% (35,804)	- reference -
Male	46.6% (1,958,814)	1.6% (30,849)	1.51 (1.50, 1.53)
Race/Ethnicity			
Non-Hispanic white	62.9% (2,646,411)	1.4% (36,008)	- reference -
Non-Hispanic Black or African American	5.2% (217,992)	5.1% (11,223)	3.93 (3.88, 3.99)
Non-Hispanic American Indian, Alaska Native, Native Hawaiian or Other Pacific Islander	0.5% (20,651)	2.9% (605)	2.13 (2.02, 2.25)
Non-Hispanic Asian or Indian	0.2% (7,115)	0.6% (41)	0.42 (0.33, 0.54)
Hispanic of any race	13.6% (570,177)	2.4% (13,447)	1.68 (1.65, 1.70)
Unknown	17.7% (743,388)	0.7% (5,333)	1.08 (1.05, 1.10)

**Fig. 1:**
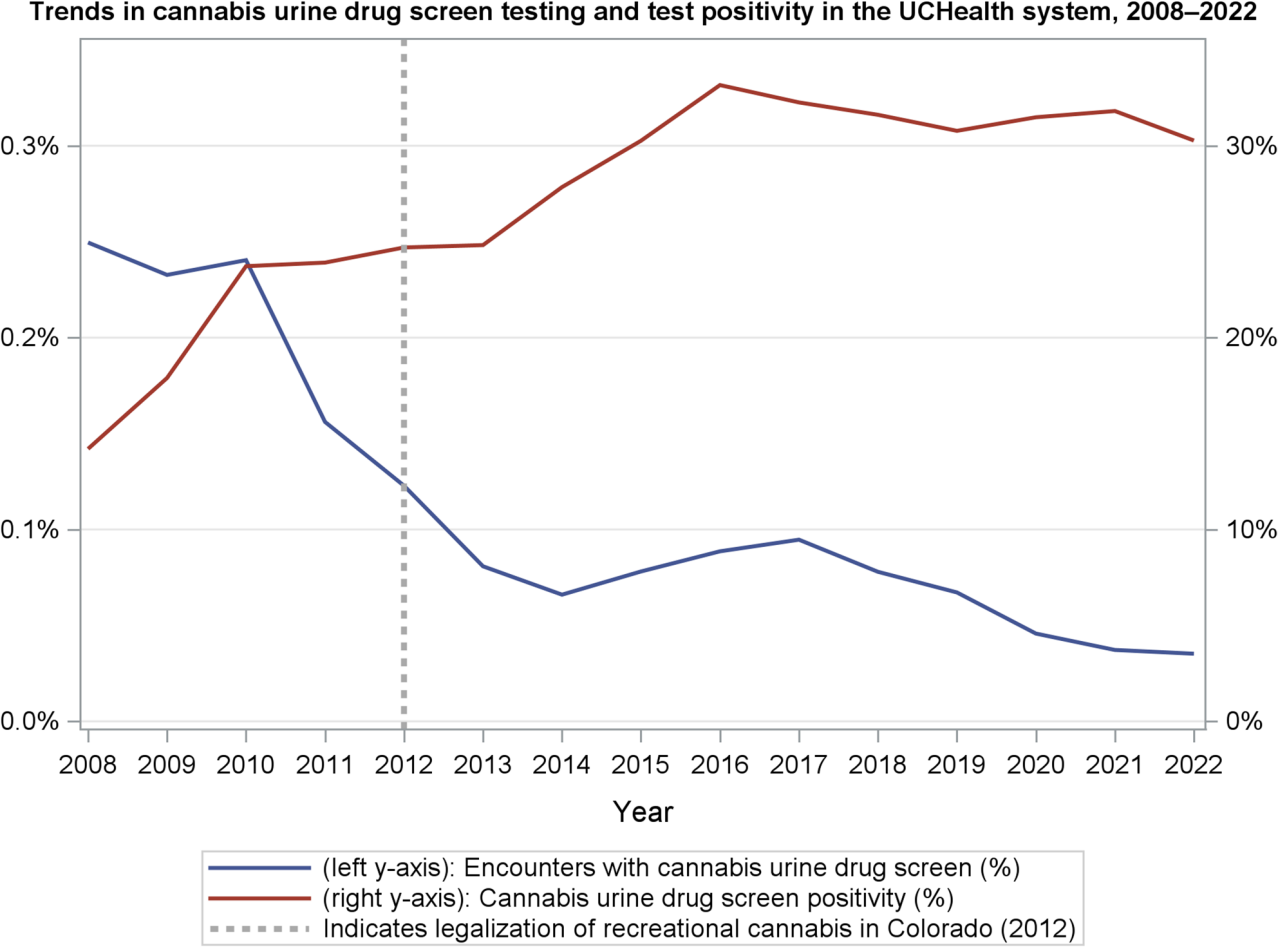
Annual trends in the percentage of UCHealth encounters that included a cannabis urine drug screen (left y-axis, blue line) and the percentage of cannabis urine drug screens that were positive (right y-axis, red line) from 2008 to 2022. The vertical dashed line indicates the year (2012) in which recreational cannabis was legalized in Colorado

Cannabis urine drug screen (UDS) testing occurred in a small percentage of encounters overall during the study period; the percentage of encounters that included cannabis UDS testing declined over time, decreasing from 0.25% in 2008 to 0.04% in 2022 (Fig. 1). The percentage of cannabis UDS that were positive increased from 14% in 2008 to 30% in 2015, after which positivity rates plateaued.

Testing rates varied by patient demographics, as seen in multivariable models (Table 1). Patients under 18, 18–39, 40–59, and 60–79 were more likely to be tested compared to patients 80 years and older ([95% CI] 5.89 [5.59, 6.20], 13.70 [13.11, 14.32], 8.63 [8.25, 9.02], and 3.78 [3.62, 3.96], respectively, with the highest testing rates among those aged 18–39. Males were more likely to be tested at a given visit compared to females (RR [95% CI] 1.51 [1.50, 1.53]). Racial and ethnic disparities were also noted in cannabis testing. Patients who were Black, Hispanic, and American Indian (RR [95% CI] 3.93 [3.88, 3.99], 1.68 [1.65, 1.70], and 2.13 [2.02, 2.25], respectively) were more likely to be tested than white patients at a given visit, while Asian patients were least likely (RR [95% CI] 0.42 [0.33, 0.54]). In interaction models, risk ratios for testing remained similar for a given demographic group over time, with racial and ethnic disparities persisting into the most recent 2018–2022 data (Supplementary Table 1).

## Discussion

In this longitudinal analysis of cannabis urine drug screen (UDS) testing within Colorado’s largest integrated healthcare system, we observed several key findings. First, cannabis UDS positivity increased over time following legalization and subsequently plateaued, while the frequency of cannabis UDS testing declined substantially. Second, cannabis UDS testing varied by age and sex, with higher testing rates among younger patients and males. Third, persistent racial and ethnic disparities in cannabis UDS testing were observed, despite population-based data demonstrating similar cannabis use prevalence across racial and ethnic groups.

Prior studies examining the impact of cannabis legalization in Colorado have primarily focused on healthcare utilization and acute clinical presentations. In contrast, system-wide data describing longitudinal trends in clinician-driven cannabis UDS testing are limited. The observed decline in cannabis testing over time may reflect reduced perceived clinical utility of routine cannabis screening as cannabis use became legalized and more socially normalized, as well as evolving institutional testing practices and the implementation of telehealth visits. Additionally, the healthcare system expanded to include locations in Wyoming and Nebraska where cannabis is not legal.

Differences in cannabis UDS testing by age and sex may reflect variation in clinical indications for testing, including higher rates of trauma, psychiatric evaluation, and substance-related assessments among younger patients and males. However, the persistence of racial and ethnic disparities in testing is notable. Consistent with prior studies, we observed that Black, Hispanic, and American Indian patients were more likely to undergo cannabis UDS testing than White patients, despite evidence that differences in cannabis use prevalence do not fully explain these disparities. These findings raise concern that cannabis UDS testing may not be applied uniformly across patient populations and underscore the importance of examining clinical decision-making surrounding substance-related screening and institutional policies. At present time, there appears to be a gap in the literature regarding cannabis testing on other demographics beyond race and ethnicity.

This study has several limitations. First, during the study period, the UCHealth system expanded rapidly from one to fourteen hospitals, added numerous departments, extended its reach to rural areas, and into states in which cannabis is not legal. As a result, the patient population changed over time, and we were unable to assess testing trends beyond the overall cohort. Also, we are unable to analyze populations of patients who are not served by the UCHealth system. We did not have encounter-level geographic data to determine the specific state in which each UDS was obtained; however, system-level data indicates that approximately 5% of patients served resided outside of Colorado. While this proportion is small, inclusion of patients from neighboring states where cannabis laws differ may influence the interpretation of legalization-related trends.

Second, UCHealth began using a unified electronic medical record system in 2010, which may have influenced the way visits, encounters, and tests were coded. This transition, along with system expansion, may have contributed to changes in encounter volume and the observed decline in the proportion of visits with cannabis UDS testing. With that in mind, we focused only on system-wide longitudinal trends rather than differences in specific clinical settings, such as emergency room, inpatient care, or clinic visits. Finally, UDS testing at UCHealth are ordered based on the discretion of the individual provider caring for the patient and within clinical situations where substance use may be a contributing factor to an acute or chronic condition. To this end, we could not analyze the clinical indications for which a UDS order was placed, which could be an interesting area of future research.

## Conclusions

In a large integrated healthcare system spanning the period before and after recreational cannabis legalization, cannabis urine drug screen testing declined substantially despite increasing rates of test positivity. Differences in testing by age, sex, and race/ethnicity persisted throughout the study period. These findings suggest that cannabis-related screening practices may not be applied uniformly across patient populations. Future research should examine clinical indications and institutional policies guiding cannabis UDS testing and evaluate how test results influence patient care.

## Supplementary Material

Supplementary Material 1. Supplementary Table 1: Association between patient demographics and cannabis urine drug screen testing among UCHealth patients, 2008–2022, showing trends over time from multivariable interaction models.

## Data Availability

The datasets during and/or analyzed during this study can be made available upon reasonable request.
