# Supplementary material for: Trends in cannabis urine drug screen testing in Colorado’s largest health system: a retrospective cohort study
Source: J Cannabis Res. 2026 Mar 3;8:49. doi: 10.1186/s42238-026-00418-8 (PMC13063531; doi:10.1186/s42238-026-00418-8)
Supplement: Supplementary file 1 — Supplementary Material 1. Supplementary Table 1: Association between patient demographics and cannabis urine drug screen testing among UCHealth patients, 2008–2022, showing trends over time from multivariable interaction models. [file 42238_2026_418_MOESM1_ESM.docx]

**Supplementary Table 1: Association between patient demographics and cannabis urine drug screen testing among UCHealth patients, 2008–2022, showing trends over time from multivariable interaction models**

|  | **Adjusted RR (95% CI)**  **2008-2012** | **Adjusted RR (95% CI)**  **2013-2017** | **Adjusted RR (95% CI)**  **2018-2022** |
| --- | --- | --- | --- |
| **Age** |  |  |  |
| Under 18 | 8.14 ( 7.62, 8.71) | 9.29 ( 8.45, 10.22) | 5.21 ( 4.87, 5.57) |
| 18-39 | 17.25 ( 16.38, 18.16) | 23.56 ( 21.69, 25.58) | 11.27 ( 10.63, 11.94) |
| 40-59 | 10.43 ( 9.90, 10.98) | 14.07 ( 12.95, 15.28) | 6.37 ( 6.01, 6.75) |
| 60-79 | 4.24 ( 4.03, 4.47) | 5.37 ( 4.94, 5.84) | 3.14 ( 2.96, 3.34) |
| 80+ | -reference- | -reference- | -reference- |
| **Sex** |  |  |  |
| Female | -reference- | -reference- | -reference- |
| Male | 1.62 ( 1.60, 1.64) | 1.62 ( 1.59, 1.65) | 1.37 ( 1.35, 1.39) |
| **Race/Ethnicity** |  |  |  |
| Non-Hispanic white | -reference- | -reference- | -reference- |
| Non-Hispanic Black or African American | 3.38 ( 3.33, 3.44) | 4.10 ( 4.01, 4.20) | 3.38 ( 3.31, 3.45) |
| Non-Hispanic American Indian, Alaska Native, Native Hawaiian or Other Pacific Islander | 2.14 ( 2.00, 2.28) | 2.10 ( 1.90, 2.32) | 2.13 ( 1.98, 2.29) |
| Non-Hispanic Asian or Indian | 0.58 ( 0.42, 0.79) | 0.60 ( 0.35, 1.03) | 0.41 ( 0.31, 0.56) |
| Hispanic of any race | 1.63 ( 1.60, 1.66) | 1.73 ( 1.68, 1.77) | 1.67 ( 1.64, 1.70) |
| Unknown | 1.00 ( 0.97, 1.02) | 1.18 ( 1.13, 1.23) | 0.98 ( 0.95, 1.01) |
